# Specific Proteins in Nontuberculous Mycobacteria: New Potential Tools

**DOI:** 10.1155/2015/964178

**Published:** 2015-05-28

**Authors:** Patricia Orduña, Antonia I. Castillo-Rodal, Martha E. Mercado, Samuel Ponce de León, Yolanda López-Vidal

**Affiliations:** ^1^Programa de Inmunología Molecular Microbiana, Departamento de Microbiología y Parasitología, Facultad de Medicina, Universidad Nacional Autónoma de México, 04510 Mexico City, DF, Mexico; ^2^División de Investigación Clínica, Facultad de Medicina, Universidad Nacional Autónoma de México, 04510 Mexico City, DF, Mexico

## Abstract

Nontuberculous mycobacteria (NTM) have been isolated from water, soil, air, food, protozoa, plants, animals, and humans. Although most NTM are saprophytes, approximately one-third of NTM have been associated with human diseases. In this study, we did a comparative proteomic analysis among five NTM strains isolated from several sources. There were different numbers of protein spots from *M. gordonae* (1,264), *M. nonchromogenicum* type I (894), *M. nonchromogenicum* type II (935), *M. peregrinum* (806), and *M. scrofulaceum/Mycobacterium mantenii* (1,486) strains, respectively. We identified 141 proteins common to all strains and specific proteins to each NTM strain. A total of 23 proteins were selected for its identification. Two of the common proteins identified (short-chain dehydrogenase/reductase SDR and diguanylate cyclase) did not align with *M. tuberculosis* complex protein sequences, which suggest that these proteins are found only in the NTM strains. Some of the proteins identified as common to all strains can be used as markers of NTM exposure and for the development of new diagnostic tools. Additionally, the specific proteins to NTM strains identified may represent potential candidates for the diagnosis of diseases caused by these mycobacteria.

## 1. Introduction

Mycobacteria that are not members of the* Mycobacterium tuberculosis* complex (*Mycobacterium africanum*,* Mycobacterium bovis*,* Mycobacterium bovis* BCG,* Mycobacterium canettii*,* Mycobacterium microti,* and* Mycobacterium tuberculosis*) or leprosy are classified as nontuberculous mycobacteria (NTM). The NTM group comprises more than 150 species that are widely distributed in many different environments (http://www.bacterio.cict.fr/m/mycobacterium.html). NTM have been isolated from water, soil, air, food, protozoa, plants, animals, and humans [1, 2]. Although most NTM are saprophytes, approximately one-third of NTM have been associated with human diseases [3, 4].

NTM infection is relatively uncommon and they are more frequently observed in immunocompromised individuals [5]. However, the rate of disease caused by NTM in individuals without abnormalities that would predispose them to infection appears to be increasing [6]. The development of new epidemiological tools, which are based on molecular techniques, has allowed an increased diagnosis of NTM disease and an increase in the identification of NTM species that are responsible for disease [4, 7].

Person to person transmission of NTM has not been reported, and it is generally accepted that NTM infections are acquired from environmental sources (water, soil) and that they are responsible for many nosocomial infections and occupational diseases. Some authors have indicated that NTM infections were directly related to exposure to contaminated water as they demonstrated to isolate the same clones from the water and patients [1, 8, 9]. Nevertheless, there is also a correlation between NTM-contaminated metallic fluids and aerosols with hypersensitive pneumonitis, asthma, and bronchitis observed in metallurgical workers [10]. The NTM species associated with human diseases have been isolated from the lungs, skin, and other soft tissues. Pulmonary infection is the most common disease manifestation and is associated with an increased age of the patient, while the skin and soft tissue diseases have not been associated with age or gender [4, 7].

Previous exposure to NTM has been proposed as one of the main causes of reduced efficacy of BCG vaccination against pulmonary tuberculosis infection [11, 12]. Black et al. demonstrated that young adults living in the northern part of Malawi are immunologically reactive to NTM antigens prior to vaccination with BCG [13]. In addition, numerous animal model studies have provided evidence that exposure to NTM before the BCG vaccine application may modulate the immune response that is induced by the BCG vaccine [14, 15]. Mendoza-Coronel et al. demonstrated that* Mycobacterium avium* may be implicated in the induction of immune tolerance mechanisms, which could impact the T cell response that is induced by BCG vaccination [16].

Additionally, exposure to NTM is responsible for the low predictive value of the purified protein derivative (PPD) test. The PPD or the Mantoux reaction is the only available diagnostic tool to identify latent tuberculosis. Ideally, the PPD test could be used as a marker for tuberculosis infection, but unfortunately there is cross-reactivity between NTM infection and BCG vaccination [17–19]. Black et al. described that the IFN-gamma response to* M. tuberculosis* purified protein derivative (PPD) after vaccination was lower in individuals who reacted strongly to NTM antigens [13].

The increase of NTM infections, the variability of BCG vaccine protection, and the lack of a diagnostic tool for* Mycobacterium *species infection make it necessary to identify novel proteins that can potentially be used in the development of new vaccines and diagnostic tools against tuberculosis infection [20].

In this study, we did a comparative proteomic analysis of five different NTM strains: three were isolated from the pump water in Mexico City (*Mycobacterium gordonae*,* Mycobacterium nonchromogenicum *type II, and* Mycobacterium peregrinum*), one was isolated from human pulmonary infection (*Mycobacterium scrofulaceum/Mycobacterium mantenii*), and one was purchased (*M. nonchromogenicum *type I, ATCC 1953). We identified proteins common to all strains and specific proteins to each NTM strain. Some of the proteins that were common between the strains could be used as markers of NTM exposure.

## 2. Materials and Methods

### 2.1. Bacterial Strains

**Figure 1:**
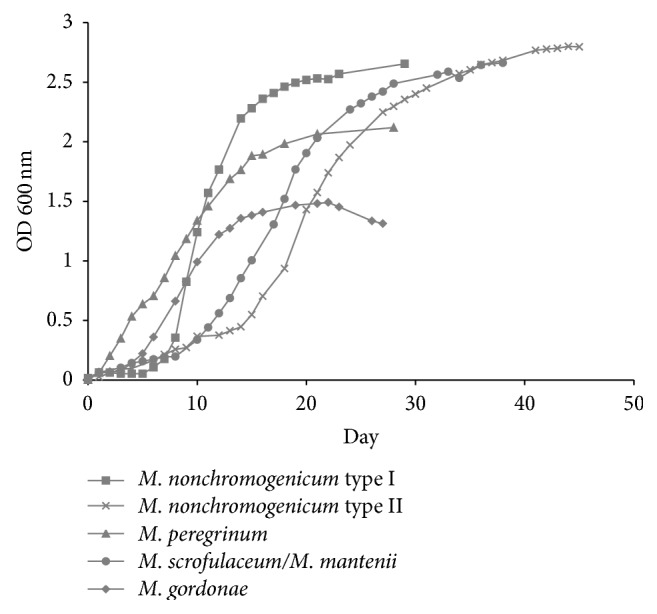
Representative growth curves of NTM strains included in this study. Growth curves were realised on Sauton medium at 37°C by duplicate.

Five strains of NTM were used in this study: three were isolated from the pump water in Mexico City as previously described by Castillo-Rodal et al. (*M. gordonae*,* M. nonchromogenicum *type II, and* M. peregrinum*) [2], one was isolated from human pulmonary infection (*M. scrofulaceum/M. mantenii*), and one was purchased (*M. nonchromogenicum *type II,* ATCC 1953*) (Table 1). To ensure that all NTM strains were in the metabolically activated state, growth curves of each strain in Sauton medium were determined (Figure 1). All strains were grown to the mid-logarithmic phase (10 days for* M. nonchromogenicum* type I, 20 days for* M. nonchromogenicum* type II, 11 days for* M. gordonae*, 8 days for* M. peregrinum*, and 17 days for* M. scrofulaceum/M. mantenii*) at 37°C with shaking and then were harvested by centrifugation, washed three times, and suspended in sterile deionised water.

### 2.2. Sample Preparation and 2D-PAGE

Cellular proteins were obtained by sonicating the bacteria (Ultrasonic Processor, Cole Parmer Corporation, USA) in the presence of protease inhibitors (10 mM PMSF, 1 mM EDTA; cycles: 1 min ON/1 min OFF) at 4°C. For 2D-PAGE, approximately 80 *μ*g of protein for the analytical gels or 100–150 *μ*g of protein for the preparative gels was solubilised, denatured, and reduced in sample buffer (4% CHAPS, 9 M urea, 70 mM l-dithiothreitol (DTT), 0.001% bromophenol blue, and 0.1% 3–10 ampholyte) and was used to rehydrate 11-cm, pH 4–7 IPG strips (ReadyStripTM, IPG strips, Bio-Rad, USA). IEF was carried out on a Multiphor II (Amersham Biosciences, UK) until 52,000 VH at 17°C. Prior to separation in the second dimension, IPG strips were equilibrated in a solution containing 6 M urea, 30% (v/v) glycerol, 50 mM Tris-base pH 8.8, and 2% (w/v) SDS. The strips were equilibrated first for 15 min with 70 mM DTT and then for 15 min with 120 mM iodoacetamide. The second-dimension electrophoresis was performed using a 12.5% polyacrylamide gel (Hoefer SE-600, Amersham Biosciences, UK) with a voltage gradient of 50–150 V for approximately four hours. Once fixed, the proteins were silver-stained and the gel images were then captured in a digital format for analysis (Molecular Imager GS-800TM Calibrated Densitometer, Bio-Rad, USA).

### 2.3. Gel Analysis and Spot Selection

2D-PAGE was performed twice for each strain, and independent cultures were utilised to eliminate technical variation. Gel analysis was performed using PDQuest-Advanced 2D Analysis V8.0 (Bio-Rad, USA). A master image gel (MIG) was integrated with the two duplicate gels of each strain and was utilised for comparison. To estimate and overcome technical variations between replicates, the spots were quantified for all of the gels. The variation in the coefficients was calculated using a previously described method [21, 22]. The spot intensity values were normalised to the total pixel count for each gel. Ten common spots for all strains, 7 specific spots for* M. gordonae* strain, and 2 specific spots for* M. arupense*,* M. nonchromogenicum, *and* M. peregrinum* strains were selected and identified by mass spectrometry. Spot selection criteria included the following: the spot was well defined, the spot had a high intensity, and the spot locations were diversely spaced throughout the gel.

### 2.4. Protein Identification

The selected spots were identified by a previously described protocol [21, 22]. Protein identification was performed using a 3200 QTRAP hybrid tandem mass spectrometer (3200 QTRAP, Applied Biosystems, USA) equipped with a nanoelectrospray ion source (NanoSpray II) and a MicroIonSpray II head. Proteins were identified based on their MS/MS spectra datasets using the MASCOT search algorithm (Version 1.6b9, Matrix Science, London, UK). A BLAST search was conducted comparing the sequences to the* M. tuberculosis* complex and Eubacteria kingdom sequences of the National Center for Biotechnology Information (NCBI) nonredundant database (NCBI nr20070623).

## 3. Results and Discussion

All of the NTM strains that were used in this study were slow growth strains, except for* M. peregrinum* that was a fast growth strain. We determined that the proteins did not have evidence of degradation by a polyacrylamide gel electrophoresis (data not shown).

**Figure 2:**
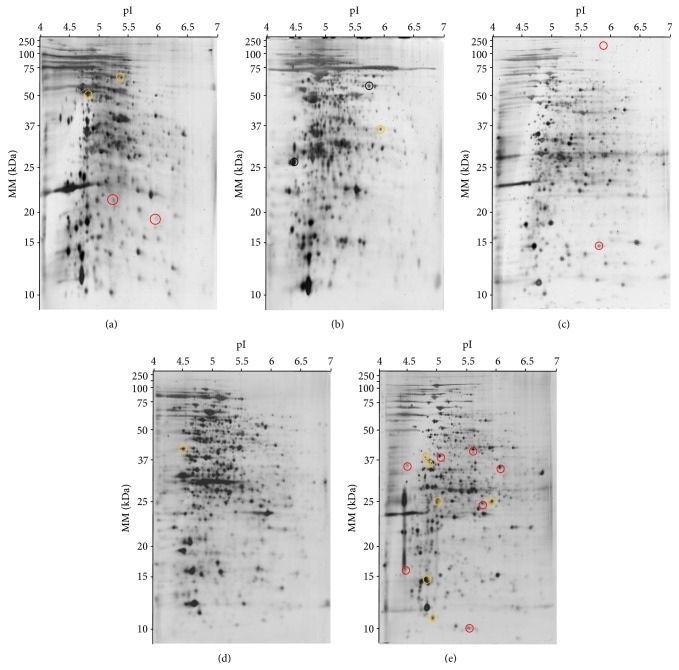
Representative 2D-PAGE of NTM cellular proteins of (a)* M. nonchromogenicum* type I, (b)* M. peregrinum*, (c)* M. nonchromogenicum* type II, (d)* M. scrofulaceum/M. mantenii*, and (e)* M. gordonae*. Eighty micrograms of cell proteins was isoelectrically focused in IPG strips (pH 4–7) and run on sodium dodecyl sulphate (SDS) 12.5% polyacrylamide gel. Gels were silver-stained and analysed with PDQuest 2D Analysis V8.0 (Bio-Rad, USA). The yellow and red circles were used to identify common proteins to all NTM strains and specific proteins to each NTM strain, respectively, by MS-based techniques.

**Figure 3:**
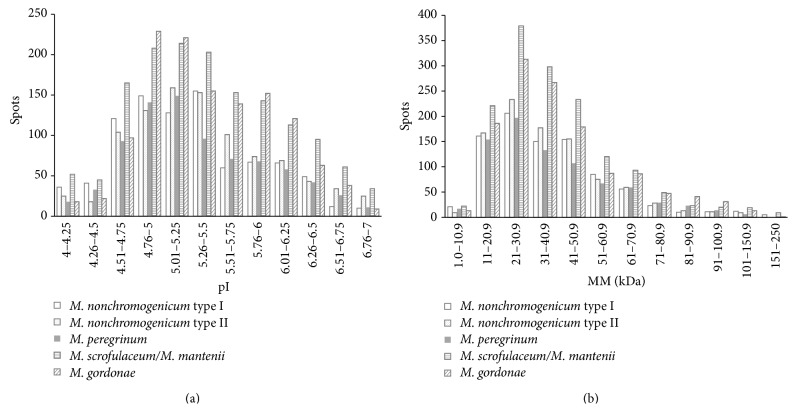
Distribution of proteins by isoelectric point (a) and molecular mass (b). Gel analysis was performed using PDQuest-Advanced 2D Analysis V8.0 (Bio-Rad, USA).

The cell fractions from the five NTM strains in the mid-logarithmic phase were isolated and then analysed by two-dimensional polyacrylamide gel electrophoresis (2D-PAGE). We identify different number of protein spots from* M. gordonae* (1,264),* M. nonchromogenicum *type I (894),* M. nonchromogenicum *type II (935),* M. peregrinum* (806), and* M. scrofulaceum/M. mantenii *(1,486) strains, respectively (Figure 2, Table 2). The distribution of the proteins by MM and pI was similar between the five NTM strains analysed (Figure 3).

Comparison of the protein profiles showed that 141 proteins were present in all NTM strains studied and approximately 80% of the proteins were shared between two or more strains (named common proteins). We also identified proteins present in only one NTM strain (named specific proteins).* M. gordonae* was observed to have the highest percentage of specific proteins, with 24% (Table 3).

A total of 23 proteins of the five NTM strains studied were selected for their identification by MS-based technologies. Spot selection criteria included the following: the spot was well defined, the spot had a high intensity, and the spot locations were diversely spaced throughout the gel.

We identify ten common proteins to all NTM strains studied (Table 4). Four of these proteins corresponded to informational pathways (RNA polymerase beta subunit, 50S ribosomal protein L7/L12, diguanylate cyclase, and DNA polymerase III), three were related to intermediary metabolism and respiration (adenylate kinase, probable aldehyde dehydrogenase, and enolase), two were identified as conserved hypothetical proteins (WAG31, Rv3075c), and one was related to lipid metabolism (short-chain dehydrogenase/reductase). The 50S ribosomal protein L7/L12, adenylate kinase, enolase, and two hypothetical proteins (WAG31 and Rv3075c) have been previously identified in the proteome of* M. tuberculosis* and* M. bovis* BCG showing that these proteins are shared with* M. tuberculosis* complex species [23–25]. Interestingly, the ribosomal protein L7/L12 and hypothetical protein WAG31 have been found in the proteome of PPD* M. tuberculosis* and/or PPD* M. bovis* [26]. Furthermore, the 50S ribosomal protein L7/L12 was described as an immunogenic protein in the BCG Mexico strain suggesting that this protein may be part of the cross-reaction observed between BCG vaccination and NTM exposure [27]. The five remaining proteins were identified for the first time in a mycobacterial proteome in this study.

The proteins of NTM that were identified in this study and have been previously described in the* M. bovis* BCG proteome can explain the cross-reactivity observed between BCG vaccination and NTM exposition. For example, the 50S ribosomal protein L7/L12, which we determined to be present in all proteomes of NTM strains studied, has been previously described as an immunogenic protein that upregulated the expression of the mannose receptor, CD80, CD86, and MHC class II molecules and it is associated with mycobacterial virulence [28, 29].

Moreover, the proteins that have previously been identified in the PPD* M. tuberculosis* proteome and that were identified in the NTM proteome, such as 50S ribosomal protein L7/L12, adenylate kinase, and hypothetical protein WAG31, may be the cause of the low predictive value of the PPD test to diagnose* M. tuberculosis* infection [26]. In fact, the 50S ribosomal protein L7/L12 is an immunogenic protein that induces a strong delayed-type hypersensitivity reaction [28], while the hypothetical protein WAG31 is involved in peptidoglycan synthesis and it has an important role in wall synthesis, cell growth, and cell division of mycobacteria [30].

Thirteen specific proteins from the NTM strains were identified by MS-based technologies (Table 4). We identified two specific proteins to* M. arupense* (deoxyuridine 5′-triphosphate nucleotidohydrolase and probable 3-hydroxyl-thioester dehydratase),* M. nonchromogenicum *(conserved hypothetical protein, catalase-peroxidase), and* M. peregrinum* (mannose-binding lectin, inositol-5-monophosphate dehydrogenase) and seven specific proteins to* M. gordonae* (probable cold shock protein A, putative mannose-specific lectin precursor, superoxide dismutase, malate dehydrogenase, F420-dependent glucose-6-phosphate dehydrogenase, luciferase-like protein, and hypothetical protein SKA58_12772). Four proteins identified as specific to* M. gordonae* (probable cold shock protein A, superoxide dismutase, malate dehydrogenase, and F420-dependent glucose-6-phosphate dehydrogenase) have also been identified in the proteomes of* M. tuberculosis *and/or* M. bovis* BCG [23–25]. Moreover, the F420-dependent glucose-6-phosphate dehydrogenase and superoxide dismutase proteins have been identified in PPD* M. avium* and* M. immunogenum* proteomes, respectively [26, 31]. Interestingly, Dong et al. described that the superoxide dismutase has one immunodominant epitope for cytotoxic T lymphocytes, which are implicated in protective immunity against tuberculosis [32]. These findings suggest that these proteins, which were identified as specific to* M. gordonae*, are shared with other NTM strains and these may be the cause of cross-reactivity against* M. tuberculosis*,* M. bovis* BCG, and/or other NTM.

On the other hand, we identified three specific proteins to* M. gordonae* (putative mannose-specific lectin precursor, luciferase-like protein, and hypothetical protein SKA58_12772) and one specific protein to* M. peregrinum* (mannose-binding lectin) that have not been previously described in any proteomic analysis of mycobacterial strains and did not align with any protein sequences in the* M. tuberculosis* complex database (data not shown). The function of these proteins is not well defined; however, we have known that the lectins can play a major role in the interaction with human cells [33, 34]. These proteins could be utilised to discriminate between NTM and* Mycobacterium tuberculosis* complex infections. However, the presence of these proteins in other NTM species that were not included in this study must be determined by specific assays.

## 4. Conclusions

NTM are a group of environmental bacteria that are considered to be potentially pathogenic both to immunocompetent and immunocompromised individuals. Exposure to these bacteria is a factor involved in the variability of the protective efficacy of the BCG vaccine because of cross-reactive antigens that are common between NTM and strains of the* M. tuberculosis* complex. Additionally, exposure to NTM is responsible for the low predictive value of the PPD test due to cross-reactivity with similar antigens.

In this study, we described the protein profiles of five NTM strains. Analysis of these profiles indicated the presence of proteins that were both common to and specific to each NTM strain. The common proteins can be utilised as markers of prior exposure to NTM. They can potentially provide a more specific diagnosis with a decreased number of false positives. Also, the proteins that were identified in the proteome of the NTM strains studied can be useful in the development of new diagnostic tools and may help explain the cross-reactivity between the PPD test and the BCG vaccination as described above.

## Supplementary Material

The analysis use bast from NCBI data base was used.

## Figures and Tables

**Table 1:** Description of the NTM strains used in this study.

Strain	Source	Growth
*M. gordonae *	Superficial water	Slow
*M. nonchromogenicum *type I (ATCC 19530)	Soil	Slow
*M. nonchromogenicum *type II	Superficial water	Slow
*M. peregrinum *	Water distribution system Mexico City	Fast
*M. scrofulaceum/M. mantenii *	Human pulmonary infection	Slow

**Table 2:** Spots identified in the 2D-PAGE gels of cellular proteins from various NTM strains.

Strain	Total spots in master image gel	CV (%)^*^
*M. gordonae *	1,264	0.2
*M. nonchromogenicum *type I	894	5.5
*M. nonchromogenicum *type II	935	0.07
*M. peregrinum *	806	0.02
*M. scrofulaceum/M. mantenii *	1,486	0.01

^*^Coefficient of variation: data were normalised according to the total density of the gel image.

**Table 3:** Common and specific proteins in the NTM strains studied.

Strain	Common proteins^*^ (%)	Specific proteins^**^ (%)
*M. gordonae *	963 (76)	301 (24)
*M. nonchromogenicum *type I	701 (81)	166 (19)
*M. nonchromogenicum *type II	723 (81)	171 (19)
*M. peregrinum *	671 (83)	135 (17)
*M. scrofulaceum/M. mantenii *	1,231 (83)	255 (17)
Proteins common to all strains	**141**	

^*^Common proteins were defined as proteins that were present in at least two NTM strains.

^**^Specific proteins were defined as proteins present only in one NTM strain.

**Table 4:** Proteins identified by sequencing in NTM strains.

Protein	Name	Function	Gene name	Peptides ID	Global score^a^	Sequence coverage %	Reference sequence	FC^b^
Proteins identified in *M. nonchromogenicum *type I								
1	Deoxyuridine 5′-triphosphate nucleotidohydrolase	Involved in the biosynthesis of thymidylate. This enzyme is involved in nucleotide metabolism Catalytic activity: dUTP + H(2)O = dUMP + pyrophosphate	*dut *	2	109	11	*M. gilvum *	7
2	Probable 3-hydroxyl-thioester dehydratase	Unknown	*htdZ *	3	65	23	*M. tuberculosis* H37Rv	7

Proteins identified in *M. nonchromogenicum *type II								
1	Conserved hypothetical protein	Unknown	—	4	33	27	*M. tuberculosis* complex	10
2	Catalase-peroxidase KATG	Multifunctional enzyme that exhibits a catalase, a broad-spectrum peroxidase, and peroxynitritase activities may play a role in the intracellular survival of mycobacteria within macrophages for the protection against reactive oxygen and nitrogen intermediates produced by phagocytic cells	*katG *	4	115	6	*M. avium *subsp*. paratuberculosis* K-10	0

Proteins identified in *M. peregrinum *								
1	Mannose-binding lectin	Unknown	*MSMEG3662 *	4	114	30	*M. smegmatis *	—
2	Inositol-5-monophosphate dehydrogenase	IMPDH catalyses the NAD-dependent oxidation of inosine 5′-monophosphate (IMP) to xanthosine 5′-monophosphate (XMP)	*PRK05567 *	3	125	6	*M. vanbaalenii *	7

Proteins identified in *M. gordonae *								
1	Probable cold shock protein A	Possibly involved in the acclimation to cold temperatures (the production of the protein is thought to be induced at low temperatures)	*cspA *	1	34	14	*M. tuberculosis* H3Rv	0
2	Putative mannose-specific lectin precursor	Lysine domain, found in a variety of enzymes involved in bacterial cell wall degradation. This domain may have a general peptidoglycan binding function	*MAB2373 *	2	72	18	*M. abscessus *	10
3	Superoxide dismutase	Destroys toxic radicals that are normally produced within the cells Catalytic activity: 2 peroxide radicals + 2H(+) = O_2_ + H_2_O_2_	*sodA *	2	81	24	*M. gordonae* and *M. assiaticum *	0
4	Malate dehydrogenase	Involved in the conversion of malate to oxaloacetate Catalytic activity: (S)-malate + NAD^+^ = oxaloacetate + NADH	*mdh *	5	197	14	*M. marinum *	7
5	Luciferase-like protein	Energy production and conversion	*Mmcs 0532 *	3	151	9	*Mycobacterium *sp.	—
6	F420-dependent glucose-6-phosphate dehydrogenase	Catalyses oxidation of glucose-6-phosphate to 6-phosphogluconolactone using coenzyme F420 (hydroxy-5-deazaflavin derivative) as the electron acceptor	*fgd *	3	148	10	*M. avium *(*M. ulcerans, M. marinum, M. chelonae*)	7
7	Hypothetical protein SKA58_12772	Predicted phosphohydrolases	*SKA58 12772 *	1	52	3	*Sphingomonas *sp. SKA58	—

Common proteins to all NTM strains								
1	RNA polymerase beta subunit	Catalyses the transcription of DNA into RNA using the four ribonucleoside triphosphates Catalytic activity: N nucleoside triphosphate = N diphosphate + [1](N)	*rpoB *	2	30	72	*M. tuberculosis *	2
2	50S ribosomal protein L7/L12	Involved in translation mechanisms. Thought to be the binding site for several of the factors involved in protein synthesis and appears to be essential for accurate translation	*rplL *	4	115	35	*Mycobacterium* sp. (*M. smegmatis *and* M. vanbaalenii*)	2
3	Adenylate kinase	This enzyme is essential in intracellular nucleotide metabolism; in addition, it has been found to act as both a nucleoside mono- and diphosphate kinase suggesting it may have a role in RNA and DNA biosynthesis Catalytic activity: ATP + AMP = ADP + ADP	*adk *	1	53	6	*M. leprae *	7
4	Short-chain dehydrogenase/reductase SDR	Involved in the fatty acid biosynthesis pathway (first reduction step) (mycolic acid biosynthesis)	*fabG *	1	47	3	*Mycobacterium *sp.	1
5	Diguanylate cyclase	Catalytic activity: 2 GTP *↔* 2 diphosphate + cyclic di-GMP	*Pat1 0480 *	1	52	2	*Pseudoalteromonas atlantica* T6c	2
6	Conserved hypothetical protein WAG 31	Unknown	*wag31 *	3	112	12	*M. tuberculosis* H37Rv, *M. avium *subsp*. paratuberculosis* K-10	10
7	Conserved hypothetical protein	Unknown	*Rv3075c *	2	65	4	*M. tuberculosis* H37Rv	10
8	Probable aldehyde dehydrogenase	Interconversion aldehyde and acid. Catalytic activity: an aldehyde + NAD+ + H_2_O = an acid + NADH	*Rv0458 *	2	41	4	*M. tuberculosis, M. bovis, M. leprae *	7
9	Enolase	Catalysing the reversible conversion of 2-phosphoglycerate into phosphoenolpyruvate. It is essential for the degradation of carbohydrates via glycolysis Catalytic activity: 2-phospho-D-glycerate = phosphoenolpyruvate + H_2_O	*eno *	4	77	10	*M. bovis* AF2122/97	7
10	DNA polymerase III (beta chain) DNAN	DNA polymerase III is a complex, multichain enzyme responsible for most of the replicative synthesis in bacteria. This DNA polymerase also exhibits 3′ to 5′ exonuclease activity. The beta chain is required for initiation of replication once it is clamped onto DNA; it slides freely (bidirectional and ATP-independent) along duplex DNA Catalytic activity: N-deoxynucleoside triphosphate = N-diphosphate + (DNA)n	*dnaN *		44	11	*M. tuberculosis *	2

^a^According to Mascot search results, protein scores >25 are significant (*P* < 0.05).

^b^FC: functional category. 0: virulence, detoxification, and adaptation; 1: lipid metabolism; 2: information pathways; 3: cell wall and cell processes; 4: stable RNAs; 5: insertion sequences and phages; 6: Pe/PPE; 7: intermediary metabolism and respiration; 8: unknown; 9: regulatory protein; 10: conserved hypothetical (from BCGList World-Wide Web Server http://genolist.pasteur.fr/BCGList).
