# Supplementary material for: Specific Proteins in Nontuberculous Mycobacteria: New Potential Tools
Source: Biomed Res Int. 2015 May 28;2015:964178. doi: 10.1155/2015/964178 (PMC4463991; doi:10.1155/2015/964178)
Supplement: Supplementary file 1 — The analysis use bast from NCBI data base was used. [file 964178.f1.pdf]

BLAST ®

Basic Local Alignment Search Tool

NCBI/ BLAST/ blastp suite/ **Formatting Results - 96S3KF5E015**
Formatting options
Download
Blast report description

ref|YP_887965| (208 letters)

RID 96S3KF5E015 (Expires on 12-20 06:17 am)

Query ID gi|118468679|ref|YP_887965.1|
Description mannose-binding lectin [Mycobacterium smegmatis str. MC2 155]
Molecule type amino acid
Query Length 208

Database Name nr
Description All non-redundant GenBank CDS translations+PDB+SwissProt+PIR+PRF excluding environmental samples from WGS projects
Program BLASTP 2.2.30+

New DELTA-BLAST, a more sensitive

Graphic Summary

Descriptions

Sequences producing significant alignments:

Description	Max score	Total score	Query cover	E value	Ident	Accession
hypothetical protein K875_04270 [Mycobacterium tuberculosis TKK-01-0051]	255	255	100%	1e-84	72%	KBZ61319.1
peptidoglycan-binding protein [Mycobacterium tuberculosis]	62.4	62.4	22%	3e-12	62%	WP_029404272.1
hypothetical protein [Mycobacterium tuberculosis]	60.5	119	23%	3e-11	61%	WP_029401579.1
hypothetical protein [Mycobacterium tuberculosis]	58.9	92.4	23%	7e-11	56%	WP_031742300.1
hypothetical protein [Mycobacterium tuberculosis]	58.5	58.5	23%	7e-11	56%	WP_031678612.1
hypothetical protein [Mycobacterium tuberculosis]	61.2	175	23%	9e-11	62%	WP_029398816.1
mycolyltransferase II [Mycobacterium tuberculosis KT-0057]	63.2	349	23%	9e-11	64%	KCG34533.1
hypothetical protein [Mycobacterium tuberculosis]	58.5	95.5	23%	1e-10	56%	WP_029401003.1
hypothetical protein [Mycobacterium tuberculosis]	58.2	58.2	23%	2e-10	56%	WP_031699806.1
hypothetical protein [Mycobacterium tuberculosis]	58.2	105	23%	2e-10	56%	WP_031748240.1

hypothetical protein [Mycobacterium tuberculosis]	58.5	58.5	22%	2e-10	62%	WP_031679078.1
hypothetical protein [Mycobacterium tuberculosis]	58.2	114	23%	3e-10	56%	WP_031677397.1
hypothetical protein [Mycobacterium tuberculosis]	58.2	156	23%	4e-10	56%	WP_031716657.1
hypothetical protein [Mycobacterium canettii]	60.8	168	26%	4e-10	59%	WP_015289732.1
hypothetical protein [Mycobacterium tuberculosis]	58.2	162	23%	5e-10	56%	WP_031737836.1
hypothetical protein [Mycobacterium canettii]	60.8	171	23%	5e-10	59%	WP_015302928.1
hypothetical protein [Mycobacterium canettii]	60.8	173	23%	5e-10	59%	WP_015292837.1
mycolyltransferase II [Mycobacterium tuberculosis BTB12-211]	60.8	229	23%	5e-10	62%	KCR30552.1
mycolyltransferase II [Mycobacterium tuberculosis KT-0025]	60.8	229	23%	5e-10	62%	KBM47586.1
hypothetical protein [Mycobacterium tuberculosis]	60.5	173	23%	5e-10	62%	WP_023642574.1
mycolyltransferase II [Mycobacterium tuberculosis TKK_02_0058]	60.8	285	23%	5e-10	62%	KBX09438.1
hypothetical protein [Mycobacterium tuberculosis]	60.5	168	23%	6e-10	62%	WP_031738033.1
hypothetical protein [Mycobacterium tuberculosis]	60.5	168	23%	6e-10	62%	WP_031683131.1
hypothetical protein [Mycobacterium tuberculosis]	60.5	168	23%	6e-10	62%	WP_031654254.1
hypothetical protein [Mycobacterium tuberculosis]	60.5	168	23%	6e-10	62%	WP_031728693.1
hypothetical protein [Mycobacterium tuberculosis]	60.5	281	23%	6e-10	62%	WP_024918601.1
hypothetical protein [Mycobacterium tuberculosis]	60.5	172	23%	6e-10	62%	WP_031725665.1
hypothetical protein [Mycobacterium tuberculosis]	60.5	172	22%	6e-10	62%	WP_031701099.1
hypothetical protein [Mycobacterium tuberculosis]	60.5	172	22%	6e-10	62%	WP_031647528.1
hypothetical protein [Mycobacterium tuberculosis]	60.5	172	22%	6e-10	62%	WP_029402188.1
hypothetical protein [Mycobacterium tuberculosis]	57.4	170	23%	1e-09	63%	WP_031695766.1
hypothetical protein [Mycobacterium tuberculosis]	57.0	110	36%	2e-09	46%	WP_031743204.1
mycolyltransferase II [Mycobacterium tuberculosis XTB13-234]	58.9	222	23%	2e-09	60%	KBS02001.1
hypothetical protein [Mycobacterium tuberculosis]	58.5	170	22%	2e-09	60%	WP_031722178.1
hypothetical protein [Mycobacterium canettii]	58.5	169	23%	3e-09	58%	WP_014000699.1
hypothetical protein [Mycobacterium tuberculosis]	58.2	167	25%	3e-09	58%	WP_029404273.1
hypothetical protein [Mycobacterium tuberculosis]	56.6	112	23%	4e-09	63%	WP_031739705.1
hypothetical protein [Mycobacterium tuberculosis]	56.2	112	23%	8e-09	63%	WP_031688871.1
mycolyltransferase II [Mycobacterium tuberculosis H3367]	57.0	283	23%	9e-09	60%	KBF26265.1

hypothetical protein GuangZ0019_1718 [Mycobacterium tuberculosis GuangZ0019]	57.0	279	23%	1e-08	63%	EQM21360.1
mycolyltransferase II [Mycobacterium tuberculosis TB_RSA38]	56.6	224	23%	1e-08	63%	KBO92989.1
hypothetical protein [Mycobacterium tuberculosis]	56.6	153	23%	1e-08	63%	WP_031661255.1
hypothetical protein [Mycobacterium tuberculosis]	56.6	113	23%	1e-08	63%	WP_024754067.1
hypothetical protein [Mycobacterium tuberculosis]	56.6	113	23%	1e-08	63%	WP_031664783.1
mycolyltransferase II [Mycobacterium tuberculosis KT-0047]	56.2	167	23%	1e-08	63%	KCG16868.1
hypothetical protein [Mycobacterium tuberculosis]	56.6	168	23%	1e-08	63%	WP_031729870.1
hypothetical protein [Mycobacterium tuberculosis]	56.6	164	23%	1e-08	63%	WP_031682455.1
hypothetical protein [Mycobacterium tuberculosis]	56.6	168	23%	1e-08	63%	WP_031734897.1
hypothetical protein [Mycobacterium tuberculosis]	56.6	168	23%	1e-08	63%	WP_031716944.1
hypothetical protein [Mycobacterium tuberculosis]	56.6	168	23%	1e-08	60%	WP_031751377.1
hypothetical protein HKBT2_1374 [Mycobacterium tuberculosis BT2]	56.6	168	23%	1e-08	63%	AHJ46171.1
MULTISPECIES: hypothetical protein [Mycobacterium tuberculosis complex]	56.6	168	23%	1e-08	63%	WP_003406625.1
hypothetical protein [Mycobacterium tuberculosis]	56.6	168	23%	1e-08	63%	WP_031729357.1
hypothetical protein [Mycobacterium tuberculosis]	56.6	168	23%	1e-08	63%	WP_031647765.1
MULTISPECIES: hypothetical protein [Mycobacterium tuberculosis complex]	56.6	113	23%	1e-08	63%	WP_010950518.1
hypothetical protein [Mycobacterium tuberculosis]	56.6	155	23%	1e-08	63%	WP_031694028.1
hypothetical protein [Mycobacterium tuberculosis]	56.6	166	23%	1e-08	63%	WP_003906571.1
hypothetical protein [Mycobacterium tuberculosis]	56.6	168	23%	1e-08	63%	WP_003904589.1
hypothetical protein [Mycobacterium tuberculosis]	56.6	168	23%	1e-08	63%	WP_031746695.1
hypothetical protein [Mycobacterium tuberculosis]	56.6	164	23%	1e-08	63%	WP_031683605.1
hypothetical protein [Mycobacterium tuberculosis]	56.6	113	23%	1e-08	63%	WP_031733083.1
hypothetical protein [Mycobacterium tuberculosis]	56.6	112	23%	1e-08	63%	WP_031739612.1
hypothetical protein [Mycobacterium tuberculosis]	56.6	168	23%	1e-08	63%	WP_031751962.1
hypothetical protein [Mycobacterium tuberculosis]	56.6	168	23%	1e-08	63%	WP_031660508.1
hypothetical protein [Mycobacterium tuberculosis]	56.6	168	23%	1e-08	63%	WP_031734369.1
hypothetical protein [Mycobacterium tuberculosis]	56.6	168	23%	1e-08	63%	WP_031657702.1
hypothetical protein [Mycobacterium tuberculosis]	56.6	107	23%	1e-08	63%	WP_019830540.1

MULTISPECIES: hypothetical protein [Mycobacterium tuberculosis complex]	56.6	168	23%	1e-08	63%	WP_003910319.1
hypothetical protein [Mycobacterium tuberculosis]	56.6	168	23%	1e-08	63%	WP_031718476.1
hypothetical protein [Mycobacterium tuberculosis]	56.6	168	22%	1e-08	60%	WP_031697158.1
hypothetical protein [Mycobacterium tuberculosis]	56.6	112	23%	1e-08	63%	WP_031725511.1
hypothetical protein [Mycobacterium tuberculosis]	56.6	163	23%	1e-08	63%	WP_029401577.1
hypothetical protein [Mycobacterium tuberculosis]	56.6	168	23%	1e-08	63%	WP_031653759.1
mycolyltransferase II [Mycobacterium tuberculosis 3280CJ]	56.6	160	22%	1e-08	63%	KBT47326.1
mycolyltransferase II [Mycobacterium tuberculosis KT-0024]	56.6	111	22%	1e-08	63%	KCF81928.1
hypothetical protein [Mycobacterium tuberculosis]	56.2	112	23%	1e-08	63%	WP_031695280.1
hypothetical protein [Mycobacterium tuberculosis]	56.6	159	22%	1e-08	63%	WP_029399667.1
mycolyltransferase II [Mycobacterium tuberculosis BTB08-022]	56.2	56.2	22%	1e-08	63%	KCO53466.1
hypothetical protein [Mycobacterium tuberculosis]	56.6	166	23%	1e-08	56%	WP_031696050.1
mycolyltransferase II [Mycobacterium tuberculosis 2100HD]	56.6	167	23%	1e-08	63%	KAN23112.1
hypothetical protein [Mycobacterium tuberculosis]	56.2	110	23%	1e-08	56%	WP_031727331.1
hypothetical protein [Mycobacterium tuberculosis]	55.8	110	22%	1e-08	63%	WP_031737597.1
hypothetical protein [Mycobacterium tuberculosis]	56.6	166	22%	1e-08	63%	WP_031686648.1
hypothetical protein [Mycobacterium tuberculosis]	56.2	85.9	22%	1e-08	63%	WP_031678613.1
hypothetical protein [Mycobacterium tuberculosis]	56.2	88.6	22%	1e-08	63%	WP_031679079.1
hypothetical protein [Mycobacterium tuberculosis]	56.2	111	22%	1e-08	63%	WP_031699807.1
hypothetical protein IQ43_03965 [Mycobacterium tuberculosis]	56.2	56.2	22%	1e-08	63%	AIH20377.1
hypothetical protein [Mycobacterium tuberculosis]	56.2	99.0	22%	1e-08	63%	WP_025686908.1
hypothetical protein [Mycobacterium tuberculosis]	56.2	56.2	22%	1e-08	63%	WP_031695282.1
hypothetical protein [Mycobacterium tuberculosis]	56.2	95.5	22%	1e-08	63%	WP_003916319.1
mycolyltransferase II [Mycobacterium tuberculosis M991]	56.2	56.2	22%	1e-08	63%	KAX16386.1
hypothetical protein [Mycobacterium tuberculosis]	56.2	56.2	22%	1e-08	63%	WP_031662455.1
peptidoglycan-binding protein [Mycobacterium tuberculosis]	51.2	51.2	18%	3e-08	59%	WP_031744498.1
mycolyltransferase II [Mycobacterium tuberculosis TB_RSA118]	54.3	54.3	22%	6e-08	61%	KCJ48237.1
hypothetical protein IU16_04040 [Mycobacterium tuberculosis]	51.6	51.6	21%	4e-07	61%	AIH63307.1
hypothetical protein IQ47_04010 [Mycobacterium tuberculosis]	50.1	50.1	20%	2e-06	60%	AIH34911.1

hypothetical protein [Mycobacterium tuberculosis]	48.5	48.5	20%	6e-06	60%	WP_031682793.1

Alignments

hypothetical protein K875_04270 [Mycobacterium tuberculosis TKK-01-0051]
Sequence ID: **gb|KBZ61319.1|** Length: 202 Number of Matches: 1
Range 1: 1 to 202

Score	Expect	Method	Identities	Positives	Gaps	Frame
255 bits(651)	1e-84()	Compositional matrix adjust.	150/208(72%)	163/208(78%)	6/208(2%)	

Features:

```
Query  1    MGDTLTAGQKLERGGSLQSGNGAYTLTLQDDGNLVLYARDKAVWSTGTNGQDVVRAEVQT  60
            M DTLT GQKL RG SL S NGAYTLTL+DDGNLVL +R +A+WST T GQ+VVRAEVQ 
Sbjct  1    MSDTLTGGQKLVRGESLASNNGAYTLTLEDDGNLVLASRGQALWSTSTEGQNVVRAEVQP  60

Query  61   DGNFVLYTAEKPVWHTDTKGKKEVKLVLQDDRNLVLYAKDGPAWSSKTDTTEPPPPAPEP  120
            DGNFVLYTA+KPVWHTDTKGKK+VKLVLQDDRNLVLYA DGPAWS+ T+T  PPPPAPE 
Sbjct  61   DGNFVLYTADKPVWHTDTKGKKDVKLVLQDDRNLVLYAADGPAWSTHTETDSPPPPAPEA  120

Query  121  EKEAEVAPAAAEEAAPAPVAEPAPAEPAPAAPPPAPPAPRTYTVVSGDTLWAIAERFYGD  180
                  A       A   VA+ APAE A    P   PA RTYTV SGDTLWAIAERFYGD
Sbjct  121  ------AADEPAAQAEEAVADEAPAEVAAEPAPEPEPAARTYTVASGDTLWAIAERFYGD  174

Query  181  GSKYQQIADASGIANPDLIHPGQVLTIP  208
            G++YQ IADASGI+NPDLI PGQVLTIP
Sbjct  175  GNRYQAIADASGISNPDLIQPGQVLTIP  202
```

peptidoglycan-binding protein, partial [Mycobacterium tuberculosis]
Sequence ID: **ref|WP_029404272.1|** Length: 53 Number of Matches: 1
Range 1: 7 to 53

Score	Expect	Method	Identities	Positives	Gaps	Frame
62.4 bits(150)	3e-12()	Compositional matrix adjust.	29/47(62%)	37/47(78%)	0/47(0%)	

Features:

```
Query  162  YTVVSGDTLWAIAERFYGDGSKYQQIADASGIANPDLIHPGQVLTIP  208
            YTVV+GDTL A+A RFYGD   Y+ IA ASGIA+PD+++ GQ L +P
Sbjct  7    YTVVAGDTLSALALRFYGDAELYRLIAAASGIADPDVVNVGQRLIMP  53
```

hypothetical protein, partial [Mycobacterium tuberculosis]
Sequence ID: **ref|WP_029401579.1|** Length: 101 Number of Matches: 2
Range 1: 56 to 101

Score	Expect	Method	Identities	Positives	Gaps	Frame
60.5 bits(145)	3e-11()	Compositional matrix adjust.	28/46(61%)	36/46(78%)	0/46(0%)	

Features:

```
Query  162  YTVVSGDTLWAIAERFYGDGSKYQQIADASGIANPDLIHPGQVLTI  207
            YTVV+GDTL A+A RFYGD   Y+ IA ASGIA+PD+++ GQ L +
Sbjct  56   YTVVAGDTLSALALRFYGDAELYRLIAAASGIADPDVVNVGQRLIM  101
```

Range 2: 4 to 51

Score	Expect	Method	Identities	Positives	Gaps	Frame
58.9 bits(141)	1e-10()	Compositional matrix adjust.	27/48(56%)	37/48(77%)	0/48(0%)	

Features:

```
Query  161  TYTVVSGDTLWAIAERFYGDGSKYQQIADASGIANPDLIHPGQVLTIP  208
            T+ VV+G+TL A+A RFYGD   Y+ IA ASGIA+PD+++ GQ L +P
Sbjct  4    THAVVAGETLSALALRFYGDAELYRLIAAASGIADPDVVNVGQRLIMP  51
```

hypothetical protein, partial [Mycobacterium tuberculosis]

Sequence ID: **ref|WP_031742300.1|** Length: 76 Number of Matches: 2
Range 1: 4 to 51

Score	Expect	Method	Identities	Positives	Gaps	Frame
58.9 bits(141)	7e-11()	Compositional matrix adjust.	27/48(56%)	37/48(77%)	0/48(0%)	

Features:

```
Query  161   TYTVVSGDTLWAIAERFYGDGSKYQQIADASGIANPDLIHPGQVLTIP  208
             T+ VV+G+TL A+A RFYGD   Y+ IA ASGIA+PD+++ GQ L +P
Sbjct  4     THAVVAGETLSALALRFYGDAELYRLIAAASGIADPDVVNVGQRLIMP  51
```

Range 2: 56 to 74

Score	Expect	Method	Identities	Positives	Gaps	Frame
33.5 bits(75)	0.098()	Compositional matrix adjust.	14/19(74%)	17/19(89%)	0/19(0%)	

Features:

```
Query  162   YTVVSGDTLWAIAERFYGD  180
             YTVV+G+TL A+A RFYGD
Sbjct  56    YTVVAGETLSALALRFYGD  74
```

hypothetical protein, partial [Mycobacterium tuberculosis]
Sequence ID: **ref|WP_031678612.1|** Length: 65 Number of Matches: 1
Range 1: 4 to 51

Score	Expect	Method	Identities	Positives	Gaps	Frame
58.5 bits(140)	7e-11()	Compositional matrix adjust.	27/48(56%)	37/48(77%)	0/48(0%)	

Features:

```
Query  161   TYTVVSGDTLWAIAERFYGDGSKYQQIADASGIANPDLIHPGQVLTIP  208
             T+ VV+G+TL A+A RFYGD   Y+ IA ASGIA+PD+++ GQ L +P
Sbjct  4     THAVVAGETLSALALRFYGDAELYRLIAAASGIADPDVVNVGQRLIMP  51
```
